# Diagnostic evaluation of suspected local anesthetic allergy in adults: a 6-year retrospective study

**DOI:** 10.1590/1414-431X2026e15704

**Published:** 2026-10-30

**Authors:** N.Y. Akçam, L. Çevirme, A.S. Yurtsever

**Affiliations:** 1 Department of Allergy and Immunology, Mersin City Training and Research Hospital, Mersin, Turkey; 2 Division of Immunology and Allergy Diseases, Adana City Training and Research Hospital, Adana, Turkey; 3 Department of Medical Pharmacology, Mersin City Training and Research Hospital, Mersin, Turkey

**Keywords:** Local anesthetics, Hypersensitivity, Drug provocation test, Skin prick test, Intradermal test, Adverse reactions

## Abstract

Although the use of local anesthetics is considered safe for minor surgical procedures, the reported frequency of adverse reactions associated with these agents remains a significant concern. Considering the administered dose of local anesthetics, it is important to recognize toxic and allergic reactions and the factors influencing them. This study aimed to evaluate the reactions in adult patients referred with suspected drug allergy, determine the frequency of true local anesthetic allergy, and investigate other types of reactions. Allergy test results were performed in 312 patients. The type, duration, and severity of the reactions were evaluated and compared with the demographic characteristics of the patients. The occurrence of reactions with the recommended alternative local anesthetic was also assessed. The results of other diagnostic tests were additionally recorded. Of the analyzed patients, 85 (27.2%) were male and 227 (72.8%) were female. The mean age was 45 years. A history of drug allergy unrelated to local anesthetics was present in 247 patients, while 65 patients (20.83%) had a history of suspected allergy to local anesthetics. Among these, lidocaine was the most frequently suspected agent in 22 patients. The specific local anesthetic agent was unknown in 30 patients. True local anesthetic allergy was confirmed in 7 of the 65 patients, while toxic epileptic seizures related to local anesthetic overdose were observed in 3 patients. In addition, true local anesthetic allergy was identified in 2 of the 247 patients with a history of drug allergy unrelated to local anesthetics. Overall, local anesthetic-related reactions were confirmed in 9 of 312 patients (2.88%), while all other test results were negative. Diagnostic testing should be performed to evaluate true allergy in patients with a history of local anesthetic reactions, and non-allergic reactions should also be considered. Drug allergies unrelated to local anesthetics may contribute to anxiety among patients and physicians. Therefore, new diagnostic algorithms are needed to evaluate not only hypersensitivity reactions but also other allergic and toxic conditions.

## Introduction

Local anesthetics are drugs that produce anesthesia at the site of application by reversibly blocking conduction in nerve fibers. The demonstration of the effectiveness of cocaine (*Erythroxylon coca*) for corneal anesthesia in ophthalmic surgery by the Austrian physician Dr. Carl Köller in 1884 marked the beginning of a revolution in medicine. However, as cocaine began to be widely used as a local anesthetic, its toxic effects became apparent, prompting efforts to develop safer alternatives. As a result, procaine, an ester-type local anesthetic, was first discovered in 1905, followed by tetracaine. Since then, local anesthetics have been widely used to provide regional anesthesia and effective analgesia in many clinical settings, particularly in dentistry, obstetrics, and minor surgical procedures (1,2). Today, local anesthetics are among the most commonly used agents in dermatology clinics, particularly for minor surgical procedures (3).

According to their chemical structure, local anesthetics are classified into two groups (esters and amides) based on the intermediate linkage between the lipophilic aromatic (benzene ring) component and the hydrophilic quaternary amine (secondary or tertiary amine) component. Ester-type local anesthetics contain an amino-ester intermediate linkage and are associated with a higher allergic potential, whereas amide-type local anesthetics contain an amino-amide intermediate linkage. In 1948, lidocaine was introduced into clinical practice as the first member of the amide-type local anesthetic group. These agents, which enable many clinical interventions and medical procedures to be performed painlessly and comfortably, exert their effects by binding to voltage-gated sodium (Na) channels and reversibly blocking cell membranes in excitable tissues in a dose-dependent manner (4-6).

Despite their widespread use worldwide, true hypersensitivity reactions to local anesthetics are very rare. Nevertheless, similar to penicillin group drugs, local anesthetics are commonly believed to frequently cause allergic reactions (7,8). There are limited data regarding the prevalence of immunoglobulin E (IgE)-mediated allergic reactions to local anesthetics. In large case series, the incidence of local anesthetic allergy has been reported to be less than 1% (0.97%). However, considering their widespread use, appropriate management of such reactions is of critical importance (9,10). Adverse reactions have been reported in 2.5-10% of patients receiving local anesthetics during dental procedures. Non-allergic reactions may present with symptoms similar to those of early-type allergic reactions. Symptoms such as dyspnea, hypotension, and syncope may occur during both allergic and non-allergic reactions, making differentiation difficult (11).

If suspected local anesthetic allergy is not confirmed, the procedure may be performed without anesthesia, or the patient may be unnecessarily exposed to the risks of general anesthesia. Suspicion of allergy may place physicians in a dilemma and lead to changes in the treatment approach. Therefore, diagnostic testing is essential. In cases of suspected reactions to local anesthetics, evaluation should include a detailed medical history, skin tests (ST), drug provocation tests (DPT), and laboratory investigations (12,13). Allergic reactions to local anesthetics include type I (IgE-mediated immediate-type) and type IV (T cell-mediated delayed-type) hypersensitivity reactions. In addition, immune complex-mediated reactions have been rarely reported (14). Type I hypersensitivity reactions may present with clinical manifestations such as urticaria, angioedema, and anaphylaxis. Type IV (delayed-type) hypersensitivity reactions may occur as late-onset swelling at the application site, localized dermatitis, or mucosal inflammation. Acute allergic contact dermatitis may develop within 48-72 h, presenting with pruritus, erythema, and localized eczematous rash, and vesicles may also be observed (15).

Local anesthetics are widely used in medical and interventional procedures; therefore, exposure to these agents is almost unavoidable. Type I hypersensitivity reactions, including anaphylaxis, have been predominantly reported as case reports involving amide-type local anesthetics (16-18). In the literature, case reports and studies from different clinics in Turkey have also reported local anesthetic hypersensitivity and local anesthetic toxicity (19-21).

## Material and Methods

This study included patients who were referred to the Allergy and Immunology Clinic of Mersin City Hospital, Turkey, between 2016 and 2022 due to a history of immediate-type reactions to any suspected drug and the need for local anesthetic use. The medical records of patients who underwent local anesthetic skin tests and drug provocation tests were retrospectively evaluated. Ethical approval was obtained from the Mersin University Ethics Committee (Decision No. 195, date: 23.03.2022). Patient data were obtained from a regularly maintained filing system in which the medical history, physical examination findings, and diagnostic test results of all patients requiring local anesthetics were recorded.

Patients younger than 18 years of age, pregnant women, and those with incomplete test results or medical records were excluded from the study. The following data were reviewed from the records: name of the suspected drug (local anesthetics and/or other drugs), medical procedure requiring anesthesia, referring clinical department, type and duration of the reported reaction, time of symptom onset, and clinical signs and symptoms involving the skin, and respiratory, cardiovascular, gastrointestinal, and central nervous systems. In addition, demographic characteristics, the presence of other drug hypersensitivity reactions, previous exposure to local anesthetics, comorbid conditions, history of atopic disease, and family history were evaluated. Data were also recorded using a standardized form that included information on whether allergic reactions developed after procedures performed with the recommended alternative local anesthetic.

Patients were classified into two groups: those with a history of local anesthetic allergy and those with a history of allergy to drugs other than local anesthetics. Skin prick tests (SPT), intradermal tests (IDT), and subcutaneous (*sc*) DPT with local anesthetics were used to investigate allergic reactions. Diagnostic tests were performed at least 4-6 weeks after the reported reaction. Patients were instructed to avoid antihistamines and antidepressants for at least 7 days prior to skin testing. The use of medications that could potentially affect test results, including adrenergic agents, calcium channel blockers, ACE inhibitors, H2 receptor antagonists, leukotriene receptor antagonists, and anti-IgE agents, was also evaluated.

In patients who reported reactions to local anesthetics, testing was performed using the suspected local anesthetic agent. In cases where the suspected agent was unknown, testing was performed using an alternative agent. Single-dose preparations without vasoconstrictors or preservatives (such as lidocaine and mepivacaine) were used. When the specific drug was unknown, testing was performed with the local anesthetic requested by the referring physician. All patients underwent SPT and IDT, followed by DPT with the tested local anesthetic agents (22-25).

Skin tests were performed on the volar surface of both forearms. Histamine hydrochloride 10 mg/mL was used as the positive control, and 0.9% NaCl (sodium chloride) was used as the negative control. Tests were applied to the volar forearm skin using the prick method with undiluted, epinephrine-free local anesthetic solutions. Skin test sites were evaluated after 15 min. For the SPT, a wheal response ≥3 mm greater than the negative control was considered positive. Patients with positive SPT results were considered hypersensitive to the tested drug, and no further testing was performed. The IDT was performed in patients with negative SPT results. The drug was injected intradermally at dilutions of 1/100 and 1/10, using 0.02-0.05 mL to produce a 2-3 mm bleb. After 20 min, an erythematous induration with a diameter ≥3 mm was considered positive, and testing was discontinued. Intradermal test solutions were prepared before testing by sterile dilution of the suspected drug in 0.9% NaCl. For DPT, allergens were freshly drawn from local anesthetic vials, which were opened no more than 2 h before the final dose administration. Only one local anesthetic was tested per day.

In patients with negative SPT and IDT results, subcutaneous (*sc*) drug provocation testing was performed on the lateral surface of the upper arm, with each dose administered to a different site. Initially, 0.1 mL was administered subcutaneously. In the absence of any local and/or systemic reaction, testing continued with subsequent doses of 0.5 and 1 mL administered at 30-min intervals. SPT is considered the gold standard for confirming IgE-mediated allergic reactions. Following subcutaneous administration, local findings, systemic symptoms, and vital signs were monitored for approximately 6 h. The test was considered positive if objective allergic symptoms (cutaneous symptoms and/or respiratory or cardiovascular manifestations) occurred within 2 h after provocation. Patients were re-evaluated after discharge to assess the development of delayed reactions.

Statistical analysis was performed using SPSS version 22 (SPSS Inc., IBM, USA). Categorical variables are reported as numbers and percentages. Continuous variables are reported as means±SD for normally distributed data, and as median and interquartile range (25th-75th percentiles) for non-normally distributed data. The chi-squared test was used to compare categorical variables. The Mann-Whitney U test was used for comparisons of non-normally distributed continuous variables, and the independent samples *t*-test was used for normally distributed continuous variables. A P-value <0.05 was considered statistically significant.

## Results

Data from 312 patients who underwent diagnostic testing with local anesthetics were analyzed. The main reason for referral was a history of drug allergy unrelated to local anesthetics in 247 patients. Sixty-five patients (20.83%) were referred due to suspected drug hypersensitivity reactions following previous procedures involving local anesthetics. Of the study population, 85 patients (27.24%) were male and 227 (72.8%) were female. The mean age was 45.55±12.29 years. Demographic characteristics according to the study groups are presented in Table 1.

**Table 1:** Demographic characteristics of study participants with suspected local anesthetic (LA) allergy.

		Non-LA hypersensitivity	LA hypersensitivity	Confirmed cases
Gender, n (%)				
Female	227 (72.8)	179 (57.37)	48 (15.38)	5 (1.6)
Male	85 (27.2)	68 (21.79)	17 (5.45)	4 (1.28)
Total	312 (100)	247 (79.17)	65 (20.83)	9 (2.88)
Age, years				
Mean±SD	45.55±12.29	45.99±12.21	43.98±12.13	42.89±8.24
Min-Max	21-75	22-75	21-69	30-58
Median	45.3	45.79	44.9	45

Among the 65 patients with suspected reactions to local anesthetics, true local anesthetic allergy was confirmed in 7 patients (10.76%) by SPT and/or DPT. In addition, 2 of the 247 patients with a history of allergy to drugs other than local anesthetics were found to have confirmed local anesthetic allergy based on test results (Figure 1). Skin tests and/or subcutaneous provocation tests were negative in the remaining 303 patients.

**Figure 1:**
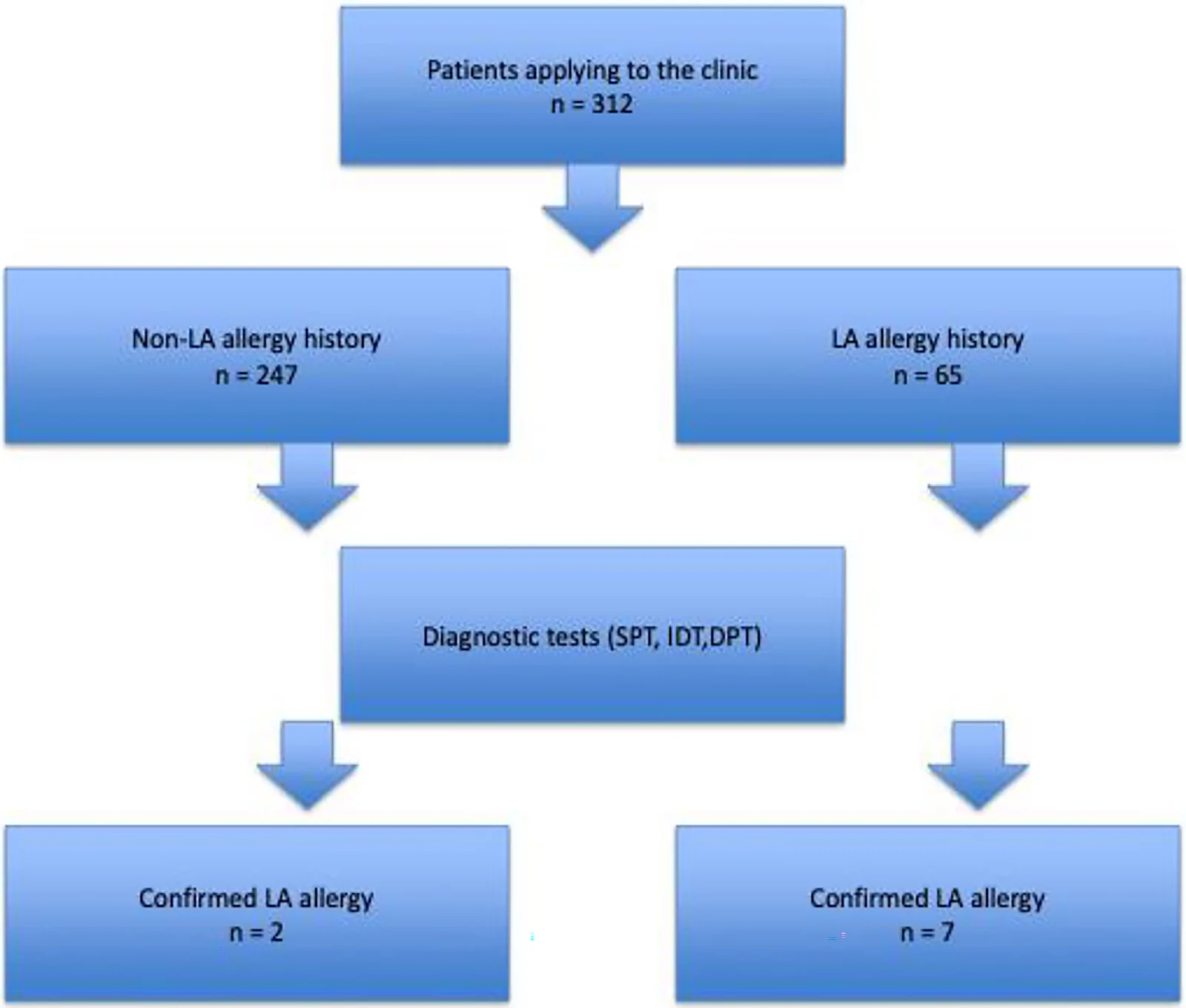
Patient flow diagram. LA: local anesthetic; SPT: skin prick test; IDT: intradermal test; PDT: drug provocation test.

Among patients referred with suspected local anesthetic allergy, 16 patients experienced reactions following local anesthetic administration without accompanying cutaneous findings such as pruritus, urticaria, or angioedema. In half of these cases, local anesthetics containing epinephrine had been used, which may increase sympathetic activity. Diagnostic tests in these patients were negative. In the remaining patients, epinephrine-free local anesthetics had been administered, and symptoms were predominantly consistent with vasovagal reactions (Table 2).

**Table 2:** General characteristics and test results of patients who developed reactions during diagnostic testing.

Confirmed diagnosis	Gender	Consulted drug hypersensitivity	Atopy	Reaction type	Tested LA	Positive test
Articaine allergy	M	ASA and metamizole		Urticaria and angioedema	Articaine, prilocaine	Articaine IDT
Articaine allergy	M	NSAID			Articaine, mepivacaine, and prilocaine	Articaine IDT
Articaine allergy	F	Articaine		Urticaria	Articaine, mepivacaine, and prilocaine	Articaine IDT
Lidocaine allergy	F	Co-trimoxazole		Anaphylaxis	Lidocaine, prilocaine	Lidocaine IDT
Lidocaine and prilocaine allergy	M	Lidocaine		Itching, dizziness	Articaine, lidocaine, mepivacaine, and prilocaine	Prilocaine IDTArticaine SPT
Prilocaine allergy	F	ASA		Urticaria	Prilocaine	Prilocaine DPT
Prilocaine allergy	F	Prilocaine		Anaphylaxis	Lidocaine, prilocaine	Prilocaine DPT
Prilocaine allergy	F	Meloxicam, dexketoprofen, naproxen	Food allergy	Anaphylaxis	Articaine, prilocaine	Prilocaine DPT
Prilocaine allergy	M	Beta-lactams, prilocaine		Anaphylaxis	Mepivacaine, prilocaine	Prilocaine DPT
Clarithromycin allergy	F	Clarithromycin-late reaction, an unknown LA-angioedema		Fixed drug eruption	Articaine	Clarithromycin DPT

M: male; F: female; LA: local anesthetic; ASA: acetylsalicylic acid; SPT: skin prick test; IDT: intradermal test; PDT: drug provocation test.

Of the 312 patients included in the study, 7 had both asthma and allergic rhinitis, and 21 had allergic rhinitis alone. Three patients had asthma only. One patient with asthma was diagnosed with nonsteroidal anti-inflammatory drug-exacerbated respiratory disease (NERD). Thirteen patients had a history of chronic spontaneous urticaria. Four patients had a history of venom allergy, and three had a history of food allergy. Overall, 52 of the 312 patients had a history of atopy. Among the 65 patients with suspected local anesthetic allergy, 11 had atopy.

When analyzed according to referring departments, the most frequent referrals were from the dentistry clinic, accounting for 252 patients (80.76%). Further results are shown in Table 3.

**Table 3:** Distribution of patients according to referring departments.

Referring Department	n (%)
Dentistry	252 (80.76)
Anesthesiology	35 (11.21)
Ear, Nose, and Throat Specialist	8 (2.56)
Obstetrics and Gynecology Specialist	6 (1.92)
Plastic and Reconstructive SurgerySelf-presented	1 (0.32)10 (3.2)

Among the 65 patients with suspected local anesthetic allergy, the suspected agent was lidocaine in 22 patients (33.85%), articaine alone in 7 patients (10.77%), prilocaine alone in 2 patients (3.08%), mepivacaine in 1 patient (1.54%), and both articaine and prilocaine in 1 patient (1.54%). In 32 patients (49.23%), the specific local anesthetic agent could not be identified (Figure 2). Additionally, 10 of these 65 patients (15.38%) had a history of allergy to both local anesthetics and other drug groups.

**Figure 2:**
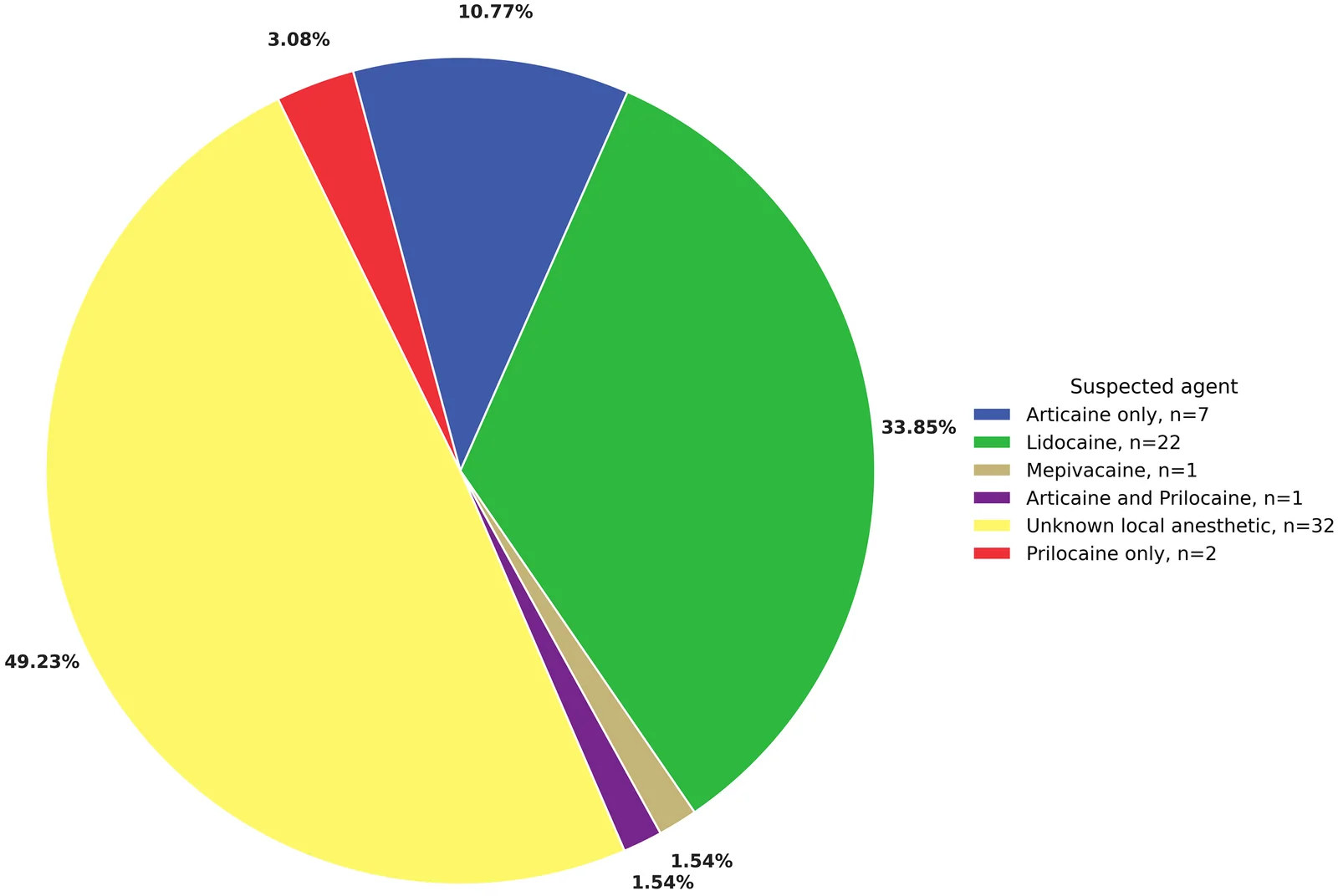
Frequency of reported reactions of local anesthetic (LA) agents in 65 patients with suspected allergy.

Diagnostic testing was performed with prilocaine in 201 patients, mepivacaine in 115 patients, articaine in 112 patients, and lidocaine in 51 patients. Overall, one patient had a positive SPT, and five patients had positive IDT results. DPTs were performed in 106 patients with articaine, 45 patients with lidocaine, 159 patients with prilocaine, and 102 patients with mepivacaine. DPT was positive in four patients tested with prilocaine only (Table 4).

**Table 4:** Results of the diagnostic tests performed and the tested local anesthetics.

LA	n	Positive diagnostic tests			Total positive tests
		SPT	IDT	DPT (n)	
Articaine (40 mg/mL)	112	0	3	0 (106)	3
Lidocaine (20 mg/mL)	51	1	1	0 (45)	2
Prilocaine (20 mg/mL)	201	0	1	4 (159)	5
Mepivacaine (30 mg/mL)	115	0	0	0 (102)	0

IDT: Intradermal test; SPT: skin prick test; DPT: drug provocation test.

Among the patients with suspected local anesthetic allergy, testing was performed with the suspected drug in 32 patients and with alternative local anesthetics in 24 patients. Local anesthetic allergy was confirmed in 7 of the 65 patients (10.76%), including allergy to prilocaine in 3 patients, articaine in 2 patients, and lidocaine in 2 patients. In addition, concomitant latex allergy, para-aminobenzoic acid (PABA) allergy, and macrolide allergy were identified in three patients. Furthermore, two patients with a history of allergy to drugs other than local anesthetics were found to have local anesthetic allergy based on test results; one tested positive for prilocaine and the other for articaine on skin testing.

In total, local anesthetic hypersensitivity was confirmed in 9 patients. The culprit agents were identified as prilocaine in 4 patients, articaine in 3 patients, and lidocaine in 2 patients. Among the 312 patients, reactions occurring after local anesthetic administration in 3 patients were evaluated as epileptic seizures due to central nervous system toxicity.

## Discussion

Since their introduction into medical practice in the late 19th century, local anesthetics have been widely used in many clinical settings, particularly in dentistry, anesthesiology, dermatology, and surgery, for purposes such as the prevention of pain during and after minor surgical procedures and the management of chronic pain (1,2). Allergic reactions to local anesthetics are known to be rare. However, the potential risks associated with their use may be greatly overestimated by both healthcare professionals and patients. Even in the absence of confirmed reactions to local anesthetics, their use during painful procedures may be unnecessarily avoided in patients with other allergic comorbidities or a history of drug allergy. Consequently, patients may be unnecessarily exposed to general anesthesia. Patients with a history of allergy to drugs other than local anesthetics or a history of reactions to general anesthetics are considered to have a higher risk of developing local anesthetic allergy (26). However, hypersensitivity was detected by diagnostic testing in only 2 of the 247 patients without a history of local anesthetic allergy.

Some studies suggest that the occurrence of unexpected adverse effects following local anesthetic administration may represent a risk factor for similar or more severe reactions upon subsequent exposure to the same agent (12,27). In nearly half of the patients referred with suspected local anesthetic reactions, adverse effects were associated with the use of epinephrine-containing preparations, supporting findings reported in the literature. Saito et al. (28) suggested that the risk of developing allergy to local anesthetics is higher in patients with a general allergic predisposition to drugs, foods, or other diseases. However, among the 65 patients referred due to suspected local anesthetic reactions, 11 had atopy and 12 had a history of allergy to other drugs. Nevertheless, local anesthetic allergy was confirmed in only 2 patients based on diagnostic test results. Among all patients, 52 had atopy and 3 had food allergy. Our findings are inconsistent with the suggestion that comorbidities, atopy, and other drug allergies increase the risk of local anesthetic allergy. This discrepancy may be explained by the relatively small number of patients with confirmed local anesthetic reactions and the imbalance in the number of patients between the groups.

Among the patients included in our study, amide-type local anesthetics had been used in 52.24% of the 65 patients with a history of hypersensitivity reactions during or after local anesthetic administration, while the specific local anesthetic agent was unknown in 47.76% of patients. Lidocaine was the most frequently suspected agent, accounting for 32.84% of cases. Amide-type local anesthetics, which are metabolized by microsomal enzymes in the liver and have longer durations of action, including mepivacaine, lidocaine, prilocaine, and bupivacaine, are preferred in clinical practice because they are associated with a lower risk of sensitization and exhibit minimal cross-reactivity. However, due to their widespread use, adverse reactions have been reported more frequently with amide-type local anesthetics (13).

Local anesthetic allergy was confirmed in only 7 of the 65 patients (10.76%) with suspected reactions. Among all cases, the total number of patients diagnosed with true local anesthetic allergy was 9 (2.88%). The findings obtained in our study are consistent with previously published data. In the literature, the reported prevalence of hypersensitivity reactions to local anesthetics ranges from 0.97 to 4.3% (13,14). Large case series confirm that the reported prevalence of local anesthetic allergy is less than 1% (0.97%) (14).

Dentistry ranks first among the disciplines in which local anesthetics are most frequently used in clinical practice. In the United States, it has been reported that approximately 384 million local anesthetic cartridges are used annually by dentists (29). Although allergic reactions related to local anesthetics are rare, their widespread use in dental practice suggests that suspected reactions may be reported more frequently following dental procedures. Consistent with this, the majority of patients referred to our clinic originated from the dentistry department, where most procedures are routinely performed under local anesthesia. The anesthesiology department ranked second among referring departments, where local anesthetics are commonly used for peripheral nerve blocks, infiltration anesthesia, neuraxial analgesia, and anesthesia. However, the absence of confirmed local anesthetic allergy in most referred patients reflects the concerns and anxiety of both physicians and patients. Increasing awareness and education in this area may help reduce unnecessary testing and alleviate concerns. Due to their widespread use, there has been a recent increase in reports of both adverse and allergic reactions. While immediate-type allergic reactions to local anesthetics are considered rare by allergy specialists, they are frequently suspected by healthcare professionals from other specialties and by patients, resulting in a large number of referrals to allergy clinics. This situation contributes to significant concern and anxiety (8,14). Since all patients were referred from clinical departments, and considering the proportion of confirmed positive results, our findings are consistent with the existing literature.

In our study, local anesthetic allergy was confirmed in 9 patients. Diagnostic tests were positive for prilocaine in five patients, articaine in three patients, and lidocaine in two patients. This finding was considered noteworthy because, when the allergy history of patients with a known suspected agent was evaluated, lidocaine ranked first (32.84%), followed by articaine (11.94%), while prilocaine ranked third (4.48%). However, this apparent discrepancy is not unexpected, given that most patients were unable to recall the specific local anesthetic responsible for their reaction. Mepivacaine ranked last among suspected agents based on allergy history, and no confirmed cases of mepivacaine allergy were identified in our study.

In two retrospective studies covering 5- and 10-year periods, none of the 326 patients with suspected immediate reactions to local anesthetics had positive provocation test results (30,31). However, it should be kept in mind that true hypersensitivity reactions to local anesthetics can occur and cannot be disregarded. A literature review conducted by Bhole et al. (14), which evaluated 23 case series involving 2,978 patients, identified 29 patients with type I hypersensitivity reactions to local anesthetics. These findings are in agreement with our results regarding both the frequency and nature of local anesthetic hypersensitivity.

Some hypersensitivity reactions to local anesthetics have been shown to be related to additives such as metabisulfite or methylparaben contained in their formulations (8). Local reactions and positive patch test results associated with sulfites (bisulfite and metabisulfite), which are added to some local anesthetic formulations to stabilize vasoconstrictors, have been reported, and this should be considered in the differential diagnosis (32). Methylparaben and propylparaben, which are used as preservatives in many local anesthetic formulations, may also cause contact dermatitis (33). Among the 65 patients with a history of suspected local anesthetic allergy, 16 did not present with cutaneous findings. In 8 of these patients, the clinical presentation was associated with the use of epinephrine-containing local anesthetics or vasovagal reactions. In the remaining 8 patients without cutaneous findings and with negative diagnostic test results, the clinical presentation was considered to be related to vasovagal reactions. In addition, the presence of paraben patch test positivity in one patient in our study is consistent with the existing literature.

Non-allergic adverse reactions include toxic reactions such as paresthesia and dizziness due to overdose, vasovagal reactions such as hypotension and sinus bradycardia, responses related to needle phobia and anxiety, including hyperventilation syndrome, and effects such as tachycardia and palpitations caused by adrenaline, which is combined with some local anesthetics to reduce absorption and prolong duration of action. These reactions account for the majority of non-allergic adverse events and are often misinterpreted as allergic reactions. In our study, three patients who were referred with a preliminary diagnosis of local anesthetic allergy were found to have experienced epileptic seizures due to central nervous system stimulation associated with systemic toxicity of local anesthetics. The suspected agents were lidocaine in one patient and prilocaine in another, while the causative agent was unknown in the third patient.

Among patients with confirmed diagnoses based on diagnostic testing, prilocaine was identified as the local anesthetic most frequently associated with anaphylaxis. In contrast, reactions associated with articaine and lidocaine were generally milder, and most patients presented with cutaneous manifestations.

## Conclusion

Local anesthetics are indispensable agents in clinical practice; however, a substantial proportion of adverse reactions associated with these drugs are related to vasovagal, toxic, or psychosomatic mechanisms rather than true allergic pathways and are frequently misinterpreted as allergic reactions. This may lead to the incorrect labeling of patients as having local anesthetic allergy, unnecessary avoidance of these agents, and, in some cases, the use of alternative procedures such as general anesthesia, which carry higher risks. As demonstrated in our study, true local anesthetic allergy is a rare condition. Furthermore, local anesthetic allergy was also found to be uncommon among patients referred due to a history of allergy to other drugs, suggesting that such a history alone may not reliably predict local anesthetic allergy.

Therefore, patients with suspected local anesthetic allergy should undergo careful evaluation, including detailed medical history and, when appropriate, skin testing and drug provocation testing. An accurate diagnostic approach is essential to prevent mislabeling, reduce unnecessary concern, and ensure the safe and appropriate use of local anesthetics.

## Data Availability

Data are available from the corresponding author upon reasonable request.
